# Developing and testing a Video assisted brief CBT intervention for children and adolescent with anxiety disorder

**DOI:** 10.1192/j.eurpsy.2023.969

**Published:** 2023-07-19

**Authors:** M. Purkayastha MUKHERJEE, S. T. Spalzang, P. Kandasamy, M. T, D. Pandian

**Affiliations:** 1 Psychiatry, JIPMER, Puducherry; 2 Psychiatry, Maanas, Salem; 3 community medicine, JIPMER, Puducherry, India

## Abstract

**Introduction:**

CBT for childhood anxiety is used as the gold treatment of choice for anxiety disorders in children. Video-assisted CBT can serve as a cost and time effective intervention method in a low-resource setting.

**Objectives:**

To develop, validate video-assisted CBT for children and adolescents with Anxiety disorder with secondary objective to explore feasibility of brief video assisted CBT as an additional component to treatment as usual in improving symptom severity.

**Methods:**

Study was divided into 2 phases. In the 1st phase videos (1 common introductory video and 2 videos each for children and adolescents,in Tamil and English) based on a validated CBT workbook was made. A second phase involving exploration of feasibility of video-based interventions along with treatment-as-usual was carried out in OPD of tertiary care hospital. Of 13 children recruited with anxiety disorder,2 were lost to follow-up. In 2nd phase, intervention delivered on OPD computer and provided to family members to watch at home through phone. Assessment of symptoms were done using SCARED, CGAS, CGI-S, VAS (parent) at baseline & 8 weeks. Written narratives were taken from participant at baseline & 8 weeks. Parent semi structured proforma was used to assess perceived benefit by parent.

**Results:**

In first phase validation was obtained from 3 experts. All experts agreed or strongly agreed for videos to be appropriate for use in children and adolescents with anxiety disorder. Most frequent diagnosis was social anxiety disorder.Family history of psychiatric illness was there in 61.54% of participants. Post intervention at 8 weeks when compared to baseline found statistically significant reduction in symptom severity on SCARED, CGAS and VAS (parent) scores. In parent semi structured proforma good improvement in understanding, perceived reduction of symptom severity reported.For qualitative data, manual content analysis done with clustering of themes and sub-themes.

In theme of Treatment impact on self, codes of decreased self-esteem and overthinking generated the maximum response. In theme of impact of illness in various contexts, codes of peer relationship and academic performance generated the maximum responses. At the end of 8 weeks participants reported Relaxation techniques as most used, followed by coping skills and challenging negative thoughts with help of tension diary respectively.

In aspects of videos liked by parents, brevity and simplicity generated most responses. In aspects requiring improvement most of parents reported no improvement needed in videos.

**Conclusions:**

This pilot study on video based CBT can serve as a time and cost-effective treatment strategy for anxiety disorders in children and adolescents especially in low resource settings. Similar studies involving development of similar videos can be made for various mental illnesses in various vernacular languages and tested in a larger population.

**Disclosure of Interest:**

None Declared

